# Divergent impacts of temperature and precipitation changes on leaf area index of deciduous broadleaf and needleleaf forests in the Northern Hemisphere

**DOI:** 10.3389/fpls.2026.1919632

**Published:** 2026-09-22

**Authors:** Rong Ma, Xiangjin Shen, Mark Henderson, Jinfeng Xu, Jiaqi Zhang, Wanying Zhou, Ming Jiang, Xianguo Lu, Binhui Liu

**Affiliations:** 1 College of Forestry, Northeast Forestry University, Harbin, China; 2 Institute of Geography and Agroecology, Chinese Academy of Sciences, Changchun, China; 3 Key Laboratory of Geographical Processes and Ecological Security in Changbai Mountains, Ministry of Education, School of Geographical Sciences, Northeast Normal University, Changchun, China; 4 Mills College, Northeastern University, Oakland, CA, United States

**Keywords:** climate change, deciduous forest, leaf area index, leaf form, Northern Hemisphere

## Abstract

**Introduction:**

Vegetation coverage significantly affects the ecological functioning of forest ecosystem. While deciduous forests with various leaf forms employ distinct ecological strategies, their vegetation coverage dynamics across the growing season and intra-seasonal periods, as well as their responses to climatic change, remain unclear. Leaf Area Index (LAI) serves as a primary parameter of physiological functional status for canopies.

**Methods:**

Using MODIS LAI data, this study analyzed variations in LAI of deciduous needleleaf forests (LAI_DNF_) and deciduous broadleaf forests (LAI_DBF_) across the Northern Hemisphere and assessed their climatic drivers.

**Results:**

This study revealed that from 2003 to 2021, LAIDBF and LAIDNF during the growing season exhibited increased rates of 0.91·10^–2^ and 0.92·10^–2^ m^2^ m^-2^yr^-1^, respectively; however, their intra-seasonal patterns differed significantly. LAI_DBF_ increased significantly in both June–August and September, whereas the significant increase in LAI_DNF_ was primarily concentrated in June–August. The increase in precipitation was associated with a general decrease in LAI for both forest types during the growing season and in varied periods; conversely, rising temperatures increased LAI across the growing season, in both May and June–August across all leaf forms. Nevertheless, it should be emphasized that the responses of LAI_DBF_ and LAI_DNF_ to temperature changes in September diverged to some extent: warming was positively associated with an increased LAI_DBF_ but showed no statistically significant change in LAI_DNF_. Interestingly, we identified asymmetric associations of LAI with diurnal warming: warming during the day had a notably more pronounced positive impact on both LAI_DBF_ and LAI_DNF_ in most regions, whereas the response of LAI_DBF_ to nighttime warming showed spatial heterogeneity. Specifically, nighttime warming was positively associated with LAI_DBF_ in areas with favorable thermal conditions (eastern United States), but negatively associated with LAI_DBF_ in cold, high-latitude regions (northeast China).

**Discussion:**

Our findings highlight the seasonally distinct responses of two deciduous forest types to climatic change and suggest that the asymmetric impacts of diurnal warming on different leaf forms should be incorporated into forest canopy leaf area dynamic prediction models.

## 1 Introduction

Forests play an irreplaceable role in regulating atmospheric greenhouse gas concentrations and mitigating global climate warming (Mo et al., 2023; Pan et al., 2024). Forest vegetation coverage can not only accurately reflect vegetation growth dynamics but also effectively influence the biogeochemical (water and carbon) cycles and energy balance processes (Kang et al., 2016; Wang et al., 2025). Over recent years, changing climates around the world have had significant impacts on the vegetation cover of many forest regions, directly affecting those forests’ canopy functioning (Zhao et al., 2021).

Deciduous forests, an important forest type, are characterized by pronounced seasonal and interannual variation patterns (Axelrod, 1966). Due to the close relationship between the phenological cycle of their canopies (from spring leaf unfolding to autumn leaf fall) and seasonal environmental fluctuations (Kikuzawa, 1991; Fu et al., 2015; Park and Jeong, 2021), they are widely regarded as an ideal index of climatic dynamics. Previous research has found that variation in deciduous forest cover is often considered a key factor in assessing forests’ carbon sequestration potential, as it directly affects the photosynthesis process by regulating the absorption of tree canopy’s photosynthetic radiation capture (Lee et al., 2023). Understanding the dynamics of deciduous forest cover and how it responds to climate change is essential for formulating scientific management policies.

The Leaf Area Index (LAI), expressed as the total one-sided leaf surface area per unit land area (Asner et al., 2003; Fang et al., 2019), offers a reliable measure of leaf abundance and spatial canopy coverage, effectively capturing variations within deciduous forest cover. As global warming intensifies, it is critical to elucidate the dynamic patterns of forest LAI and their climatic drivers. Up to now, research has mostly emphasized the variations in LAI of deciduous forests. Specifically, Zhu et al. (2016) demonstrated that global forest LAI exhibits a continuous greening trend during the growing season. Nevertheless, some research also emphasizes that this trend is characterized by significant spatial heterogeneity (Liu et al., 2012; Ma et al., 2023). Research has identified important relationships between deciduous forest LAI and climatic factors. For instance, a five-year *in situ* observation of a mixed forest composed of *Quercus frainetto* and *Quercus cerris* revealed that both the canopy coverage and chlorophyll content in these forests were significantly and positively influenced by temperature (Stagakis et al., 2022). However, researchers also suggest that (Li et al., 2023), although Larch leaf cover in high-altitude regions is predominantly driven by temperature, the advancement of phenology caused by warming has weakened their growth potential.

Precipitation is another key climatic factor regulating forest LAI dynamics (Zhang et al., 2002; Tao et al., 2020). Research indicates that, across moisture-stressed regions, precipitation patterns often serve as the primary limiting factors for LAI variations. For instance, Li et al. (2022) noted that drought has led to vegetation browning in the dry-wet climate transition zones, characterized by a significant decline in LAI. This conclusion was further supported by subsequent observations: one study indicated that, although the forest coverage rate in China increased slightly throughout this period, the increase in forest LAI was mainly concentrated in regions with better precipitation conditions, pointing to water availability as a key driver of forest canopy dynamics (Hu et al., 2021). However, a critical shortcoming of existing research lies in its emphasis on annual or whole growing season mean values for the assessment of deciduous forests cover, which effectively masks meaningful intra-seasonal differences that may exist in how these forests respond to climatic factors. Therefore, exploring changes in the LAI of deciduous forests and their climatic drivers across different intra-seasonal periods within the growing season is key to revealing their unique ecological processes.

Leaf form is an important indicator for characterizing the functional traits of trees (Bond, 1989; Becker, 2000), with needleleaf and broadleaf forms profoundly reflecting functional trade-offs in terms of photosynthesis, respiration, transpiration, and hydraulic conduction (Munier et al., 2018). While most deciduous trees display the broadleaf form, deciduous needleleaf trees are found across the Northern Hemisphere, including *Larix*, *Metasequoia*, and *Taxodium* species; the physiological differences represented by leaf forms reflect their distinct environmental adaptation strategies (Kikuzawa, 1991; Ma et al., 2023). Although existing research has shown that the two differ in phenology and their climatic response (Piao et al., 2006; Deng et al., 2025), the specific response patterns of their LAI changes to climate factors have not yet been clearly explained. Accordingly, additional analysis is essential to systematically characterize large-scale LAI dynamics for these distinct leaf forms and identify their key climatic drivers. Simultaneously, as the globe is currently experiencing asymmetric diurnal warming, existing studies have documented heterogeneous impacts of diurnal temperature variations on forest dynamics (Deng et al., 2019; Shi et al., 2023; Lv et al., 2024). However, vegetation responses to this asymmetric warming are not monotonic but are highly modulated by seasonality, water availability, and radiation (Tan et al., 2015; Piao et al., 2020; Cox et al., 2020). Previous studies have collectively shown that the responses of vegetation and LAI to diurnal climate variations are critically modulated by seasonality, water availability, thermal thresholds, and atmospheric radiation and moisture demand (Wu et al., 2016; Liu et al., 2023; Tao et al., 2022; Cox et al., 2020). Although advanced models have been developed for accurate LAI predictions (e.g., Jiang et al., 2024), how these seasonally modulated and moisture-radiation-constrained asymmetric thermal effects systematically shape the LAI differences between the two major leaf forms (needleleaf versus broadleaf) in Northern Hemisphere deciduous forests remains unclear. Further investigation is needed to determine whether asymmetric warming has divergent influences on the two leaf types of deciduous forests—nor whether such effects exhibit seasonal heterogeneity.

This study is designed to clarify the dynamics of LAI of deciduous forests and their climatic drivers (including daytime and nighttime temperature changes), using climate data and satellite-derived LAI MODIS data in the Northern Hemisphere during 2003–2021. This study aims to: (1) quantify LAI dynamics of deciduous forests; (2) compare the LAI variations and climatic drivers among different leaf forms (needleleaf and broadleaf) across the growing season and within intra-seasonal periods; (3) reveal the respective roles of daytime versus nighttime warming on LAI of deciduous needleleaf and broadleaf forests.

## 2 Materials and methods

### 2.1 Datasets

This study adopted the Leaf Area Index (LAI) data from the MCD15A3H.061 product, provided by the National Aeronautics and Space Administration (NASA) Land Processes Distributed Active Archive Center (https://lpdaac.usgs.gov/products/mcd15a3hv061). This product is jointly derived from the Terra and Aqua MODIS sensors, featuring global coverage under the MODIS Sinusoidal tiled grid system with a spatial resolution of 500 m and a consistent 4-day temporal resolution. The compositing algorithm selects optimal cloud-free observations within each 4-day window to minimize contamination from clouds, aerosols, snow, and view-angle effects. In the preprocessing phase, we performed a rigorous pixel screening based on the embedded quality assessment layer, masking all pixels flagged as low-quality, cloud-covered, or snow-covered, and identifying fill values (e.g., 255, which correspond to water bodies, bare soil, clouds, and other invalid areas) to mitigate the impacts of atmospheric and surface noise on LAI retrievals. For pixels with missing monthly values, we applied linear interpolation only when the data gap was ≤ 8 days; gaps longer than 8 days were left as missing to avoid introducing artifacts from extended interpolation.

We employed the 500 m resolution annual global land cover product MCD12Q1 (Version 6) for the period 2001–2020, distributed by the LP DAAC (https://www.earthdata.nasa.gov/data/catalog/lpcloud-mcd12q1-006). Each HDF-EOS2 granule provides six independent classification schemes. This study adopts the IGBP layer (LC_Type1) as the primary reference for forest type identification. According to the IGBP classification, deciduous needleleaf forests (DNF, class 3) and deciduous broadleaf forests (DBF, class 4) are defined as woody vegetation with a canopy height greater than 2 m and tree cover exceeding 60 %. Additionally, the pixel-level quality control (QC) band embedded in each granule was utilized to screen out unreliable observations during subsequent preprocessing.

We used the MCD12Q2 Version 6.1 annual global land cover dynamics phenology product, distributed by the LP DAAC (https://lpdaac.usgs.gov/products/mcd12q2v061). This dataset provides consistent vegetation phenology retrievals at 500 m spatial resolution under the MODIS Sinusoidal tiled projection, covering the period 2003–2021. All phenological metrics are derived from gap-filled time series of the two-band Enhanced Vegetation Index (EVI2) computed from MODIS Nadir BRDF-Adjusted Reflectance (NBAR). For each detected cycle, the start of the growing season (SOS) is defined as the date when the EVI2 time series first crosses 15% of the segment EVI2 amplitude, while the end of the growing season (EOS) is defined as the date when the EVI2 time series last crosses the same threshold. The growing season length is then calculated as the number of days between SOS and EOS.

With a spatial resolution of 2.5 min, the monthly climatic variables, including the maximum (T_max_), minimum (T_min_) and precipitation (P_re_) for each pixel point spanning 2003 to 2021 utilized in this study were obtained from WorldClim (https://www.worldclim.org/data/monthlywth.html). These datasets were subjected to thorough validation and have been broadly employed to assess the influence of global warming on plants (Fu et al., 2025; Zhang et al., 2025). The WorldClim data are in WGS84 geographic coordinates (EPSG:4326). For each period (growing season and intra-seasonal phases), precipitation was calculated as the cumulative sum of monthly values, while T_max_ and T_min_ were calculated as arithmetic means.

### 2.2 Methods

#### 2.2.1 Forest type extraction and preprocessing

Based on the MCD12Q1 product, deciduous needleleaf forests (DNF) and deciduous broadleaf forests (DBF) were extracted according to their IGBP classification, while all other land cover types were excluded. To eliminate the interference of land transformation dynamics, we masked the area of deciduous forests (including deciduous needleleaf forests and deciduous broadleaf forests) unchanged between 2001 and 2020 (**Figure 1a**). Here, unchanged refers to the land cover/land use type at the same spatial location being maintained as a specific vegetation type (deciduous forest in this study) across the study period and not experiencing any transition to other types throughout the study period (Wu et al., 2015; Shen et al., 2022). The overall workflow of the extraction process is illustrated in **Figure 2**. For each unchanged deciduous forest pixel identified from the MCD12Q1 mask, we extracted the corresponding SOS and EOS values from MCD12Q2. To ensure reliability, we discarded all retrievals flagged as low quality using the QC band, retaining only pixels with good confidence over the study period (2003–2021). We then calculated the multi-year average DOY of SOS and EOS separately for deciduous broadleaf forests (DBF) and deciduous needleleaf forests (DNF). The results show that DBF have a mean SOS of 117 DOY and EOS of 283 DOY, while DNF exhibit a narrower active window with mean SOS of 122 DOY and EOS of 276 DOY. To eliminate phenological phase mismatch between the two functional types, we defined a common overlapping active canopy interval from 122 DOY to 276 DOY, during which both forest types sustain leaf expansion without large-scale dormancy. This overlapping window (**Figure 2**) was adopted for all subsequent comparative analyses, consistent with the climatological May–September growing season suggested by previous studies (Xue et al., 2016; D’Orangeville et al., 2018; Hou et al., 2020; Gu et al., 2022; Tang et al., 2026).

**Figure 1:**
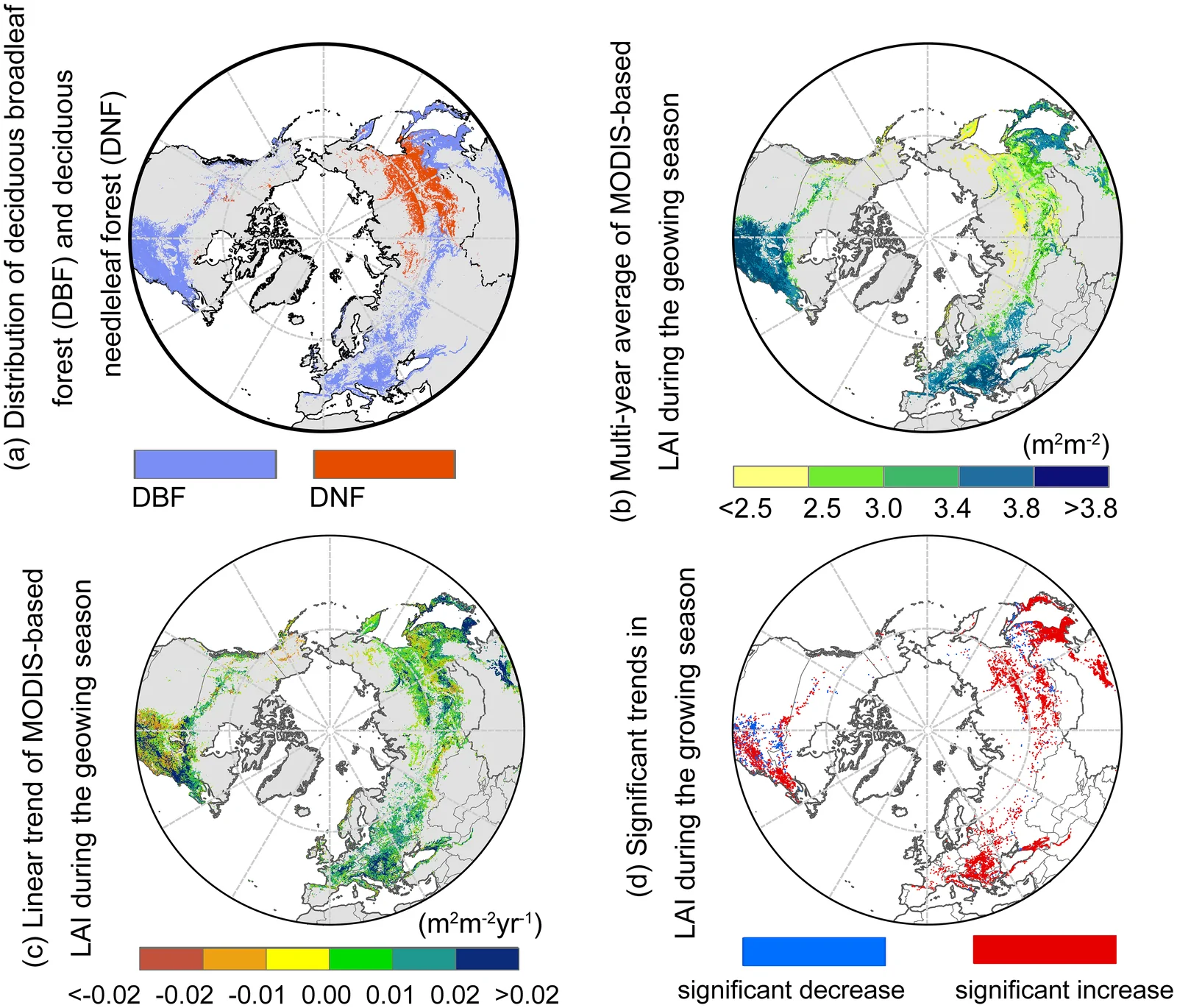
Distribution and spatial dynamics of deciduous forests during the growing season in the Northern Hemisphere from 2003 to 2021. **(a)** Distribution of deciduous broadleaf (approximately 8,463,342 pixels) and needleleaf forests (approximately 7,745,951 pixels) in the Northern Hemisphere; **(b)** Multiyear growing season average Leaf Area Index (LAI) of deciduous forests; **(c)** Linear trends in growing season LAI of deciduous forests per year; **(d)** The significant increase (red) and decrease (blue) in LAI based on FDR-adjusted significance (*q* = 0.05).

**Figure 2:**
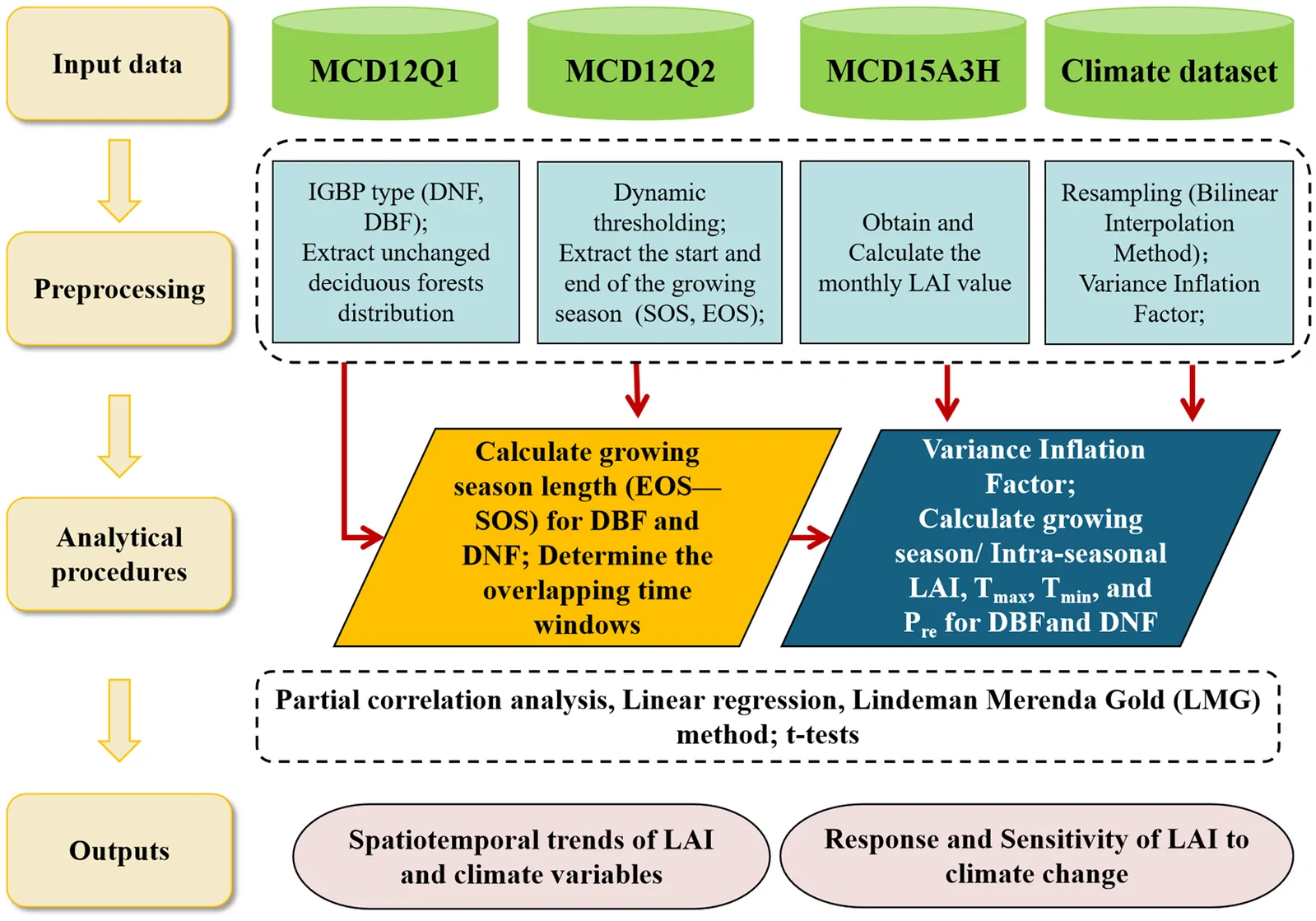
Study workflow.

Accordingly, we further divided the growing season into three intra-seasonal intervals: May, June–August, and September. The 4-day LAI data were quality-screened, and the valid values were averaged arithmetically to produce monthly LAI estimates. Subsequently, we computed the mean LAI for the entire growing season as well as for each intra-seasonal period separately. Correspondingly, LAI_DBF_ and LAI_DNF_ represent the average LAI values of deciduous broadleaf and deciduous needleleaf forests, respectively. All these abbreviations are used henceforth. We also determined the matching meteorological elements for each phase. In addition, the meteorological elements for both the growing season and individual intra-seasonal periods were resampled to a 500 m pixel resolution, consistent with that of the forest LAI. The bilinear interpolation method was selected because temperature and precipitation are continuous variables, and bilinear interpolation computes new pixel values as a weighted distance average of the four nearest input cell centers, thereby producing a smooth surface well suited for climatic variables.

#### 2.2.2 Trend analysis

To quantify the spatiotemporal variation trends of LAI_DF_ and key meteorological elements for the growing season and its constituent phases, this study computed the simple linear regression to estimate the trend (Piao et al., 2011; Zhang et al., 2025). Heteroskedasticity- and autocorrelation-consistent (HAC) standard errors were used in our regression analyses to ensure reliable statistical inference. Residual diagnostics (Q-Q plots and residual-versus-fitted plots) confirmed approximate normality and homoscedasticity. To explore how deciduous forest LAI responds to climate change, linear regression analysis of the LAI against temperature and precipitation was performed for each forest type at each pixel during 2003–2021. Numerous studies have extensively applied this approach to investigate how vegetation dynamics react to the ongoing warming of the global climate (Gu et al., 2022). The regression slopes represent the sensitivity between LAI and each climate variable (denoted as S_pre_ for precipitation, S_Tmax_ for maximum temperature, and S_Tmin_ for minimum temperature). A positive (or negative) slope indicates that LAI increases (or decreases) per unit increase in the corresponding climate variable. We employed two analytical scales. Specifically, the temporal dimension consists of 19 annual observations (2003–2021), and the spatial dimension comprises all retained deciduous forest pixels after quality control filtering (deciduous needleleaf forests: 7,745,951 pixels; deciduous broadleaf forests: 8,463,342 pixels). For spatial heterogeneity, the pixel-level regressions were used for exploratory mapping. All formal statistical inferences—including trend significance tests, partial correlations, and sensitivity estimates—were based on regionally aggregated time series, with 19 annual observations per forest type derived by arithmetically averaging all retained pixel values. This analytical design explicitly prevents spatial autocorrelation from inflating the effective sample size, because the degrees of freedom in all significance tests are determined by the number of temporal replicates.

#### 2.2.3 Detecting climate effects

To distinguish long-term trends from interannual climate-LAI covariation and account for serial correlation, we employed a multiple regression framework incorporating year and climatic variables jointly for trend attribution with HAC standard errors, and used first-differencing in partial correlation analyses as a robustness check to isolate interannual responses (yielding 18 year-to-year change observations). To ensure the robustness of the analysis and avoid potential multicollinearity among the climatic predictors, we diagnosed multicollinearity among precipitation, maximum temperature (T_max_), and minimum temperature (T_min_) using the Variance Inflation Factor (VIF). This metric quantifies the extent to which the variance of a regression coefficient is inflated due to correlations with other predictors (O’brien, 2007; Yang et al., 2025). VIF values exceeding 5 were considered indicative of moderate collinearity, while values above 10 were regarded as severe. For DBF, the VIF values were 1.62 for precipitation, 7.31 for T_max_, and 7.40 for T_min_. For DNF, the corresponding values were 2.64 for precipitation, 7.52 for T_max_, and 6.26 for T_min_. The VIF for precipitation in both forest types indicated negligible collinearity with the two temperature variables. In contrast, the VIFs for T_max_ and T_min_ ranged between 5 and 10, reflecting moderate but acceptable collinearity due to their inherent covariance. Such levels of association are common in climatic data and do not severely compromise the stability of the coefficient estimates.

Given that one of our central objectives is to examine the effects of daytime versus nighttime temperatures, we used T_max_ and T_min_ as widely adopted proxies (Peng et al., 2013; Cox et al., 2020). To further mitigate the influence of multicollinearity and isolate the independent effect of each climatic factor, we based our primary inference on partial correlation analysis (Shen et al., 2024). This approach quantifies the strength of the linear relationship between LAI and each climatic factor after removing the shared variance contributed by the other two predictors. Partial correlation analysis effectively quantifies the association among two factors while removing the confounding effects of other relevant variables (Yang et al., 2019; Han et al., 2023), thereby directly revealing the relationship between LAI and a particular climatic factor. Specifically, when estimating the correlation between precipitation and LAI, we controlled for T_max_. When analyzing the association between T_max_ (T_min_) and LAI, we controlled for precipitation. The statistical significance of the derived coefficients was assessed using t-tests and the false-discovery-rate procedure (FDR, *q* = 0.05) to evaluate the climatic influence on LAI (Forzieri et al., 2022). To further test whether the coefficients of T_max_ and T_min_ differ significantly, we fitted both variables jointly in a multiple linear regression and applied Wald tests. Simultaneously, we applied the Lindeman-Merenda-Gold (LMG) method, which partitions the total explained variance from multiple linear regression into the unique contribution of each climate variable. The statistical analyses were performed using R version 4.4.1 with packages including car (v3.1-2), relaimpo (v2.2-6), lmtest (v0.9-40), and sandwich (v3.1-0). Spatial data processing was conducted using the MODIS Reprojection Tool (MRT), and Google Earth Engine.

## 3 Results

### 3.1 Spatiotemporal variations of LAI of deciduous forests

From 2003 to 2021, the multi-year average of growing season LAI_DF_ was approximately 3.45 m^2^m^-2^. For the two types of deciduous forests, the multi-year average LAI_DBF_ and LAI_DNF_ were 3.58 and 2.34 m^2^m^-2^, respectively, both reaching their minimum values in May and peaking at June–August. Spatially, the distribution and variations of LAI_DF_ exhibited obvious regional differences (**Figure 1b**). The highest value of LAI_DF_ occurred in the eastern United States, with an overall average of over 3.80 m^2^m^-2^, while Asian high-latitude regions exhibit comparatively low average vegetation coverage (**Figure 1b**).

In addition, this study revealed an overall greening trend in LAI_DF_ at most regions across the Northern Hemisphere (**Figure 1**), with the most pronounced increases occurring at high-latitude regions (**Figure 1d**). In contrast, the variation patterns of LAI_DF_ in lower latitudes displayed considerable spatial heterogeneity, with some regions showing a declining trend (**Figure 1c**). Specifically, a significant increasing trend in LAI was observed for deciduous needleleaf forests in high-latitude regions. Notably, deciduous broadleaf forests in mid-to-high latitude Central Europe, despite starting from a lower initial value, nevertheless exhibited a similarly significant increase.

During 2003–2021, the LAI_DF_ exhibited a significant rise, with an increasing trend at 0.81·10^–2^ m^2^m^-2^yr^-1^ (*p* = 0.017), with varying degrees of significance observed across different leaf forms (**Figure 3**). The LAI_DBF_ and the LAI_DNF_ exhibited increasing trends of 0.91·10^–2^ m^2^m^-2^yr^-1^ (*p* = 0.010) and 0.92 ·10^–2^ m^2^m^-2^yr^-1^ (*p* = 0.031), respectively (**Figure 3**). Our subsequent analysis revealed that both LAI_DBF_ and LAI_DNF_ increased significantly across intra-seasonal periods: significant increasing trends during June–August (0.75·10^-2^ m^2^m^-2^yr^-1^) and September (0.82·10^-2^ m^2^m^-2^yr^-1^) were detected in LAI_DBF_, whereas for LAI_DNF_, the significant increasing trend was limited to June–August (1.20·10^-2^ m^2^m^-2^yr^-1^). However, in September, significant differences emerged: DBF still showed a statistically significant positive trend in LAI (*p* = 0.025), while DNF exhibited no statistically significant change (*p* = 0.601). DNF was mainly distributed in the high-latitude northern regions, but in most temperate regions, DBF could still maintain the upward trend of LAI. The exact cause of this late-season pattern difference, whether it is due to leaf functional strategies or the combined effect of spatial environmental conditions, requires further analysis.

**Figure 3:**
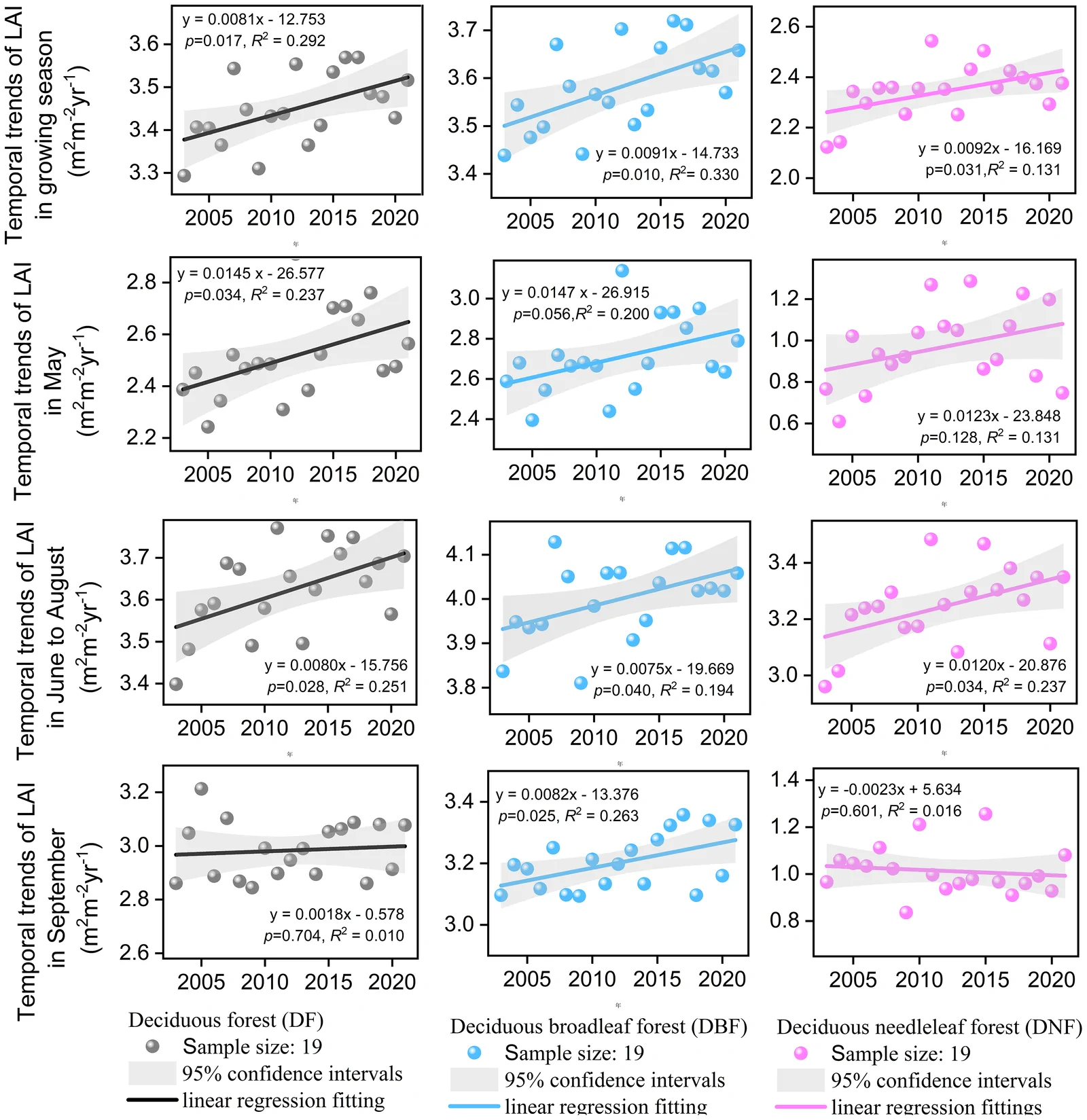
Temporal trends in Leaf Area Index (LAI) of deciduous forests (DF), deciduous broadleaf forests (DBF), and deciduous needleleaf forests (DNF) during the growing season and May, June–August, and September from 2003–2021. Solid lines represent fitted linear trends; shaded areas denote 95% confidence bands of regression estimates. Each group contains 19 annual sample observations.

### 3.2 Variations of meteorological elements in the distribution region of deciduous forests

The growing season temperatures exhibited a warming trend in high-latitude areas, while at the same time, precipitation in this region decreased (**Figure 4**), and the dominant forest type distributed in this region was mainly deciduous needleleaf forests (**Figure 1a**). From 2003 to 2021, a general increase was observed in growing season precipitation (0.08 mm·yr ^-1^), but with divergent trends across forest types: a statistically insignificant rise in deciduous broadleaf forests (0.29 mm·yr ^-1^) versus a slight decline in needleleaf forests (-0.12 mm·yr ^-1^). Concurrently, significant warming was recorded for both T_max_ and T_min_ (0.05 °C·yr ^-1^ and 0.03 °C·yr ^-1^, *p* < 0.01) in the deciduous forest area. Notably, in deciduous needleleaf forests, the increase in T_max_ (0.06 °C·yr ^-1^, *p* < 0.01) was substantially greater than that in T_min_ (0.03 °C·yr ^-1^, *p* < 0.05).

**Figure 4:**
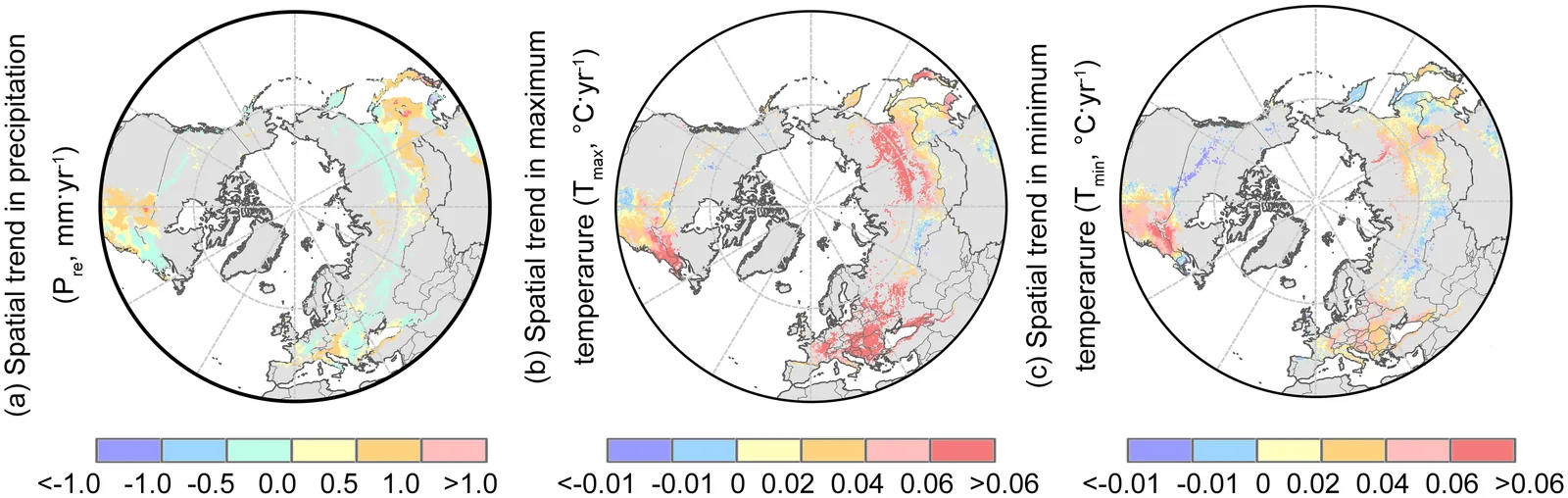
Spatiotemporal variations of meteorological factors during the growing season in the deciduous forest distribution area of the Northern Hemisphere from 2003 to 2021. **(a)** Spatial trend in precipitation (P_re_); **(b)** Spatial trend in maximum temperature (T_max)_; and **(c)** Spatial trend in minimum temperature (T_min_).

Regarding intra-seasonal temperatures (**Figure 5**), the statistically insignificant rise in T_max_ and T_min_ during May was also recorded in both deciduous broadleaf (0.02 °C·yr^-1^) and needleleaf forests regions (0.01 °C·yr^-1^). Throughout June–August, T_max_ increased significantly across all forest types, with the exception of the increase in T_min_ for deciduous needleleaf and broadleaf forests (0.03 °C·yr^-1^ and 0.03 °C·yr^-1^). T_min_ for deciduous broadleaf forests experienced significant warming in September, and the increase in T_max_ was also notable. Regarding precipitation, May showed a consistent increase trend across all forest types. In contrast, June–August and September exhibited divergent patterns: precipitation in deciduous broadleaf forests increased, though not statistically significantly (0.01 and 0.02 mm·yr^-1^, respectively), while that in deciduous needleleaf forests declined and increased (-0.37 and 0.06 mm·yr^-1^), respectively. This divergence resulted in a statistically insignificant precipitation trend for Northern Hemisphere deciduous forests overall during summer.

**Figure 5:**
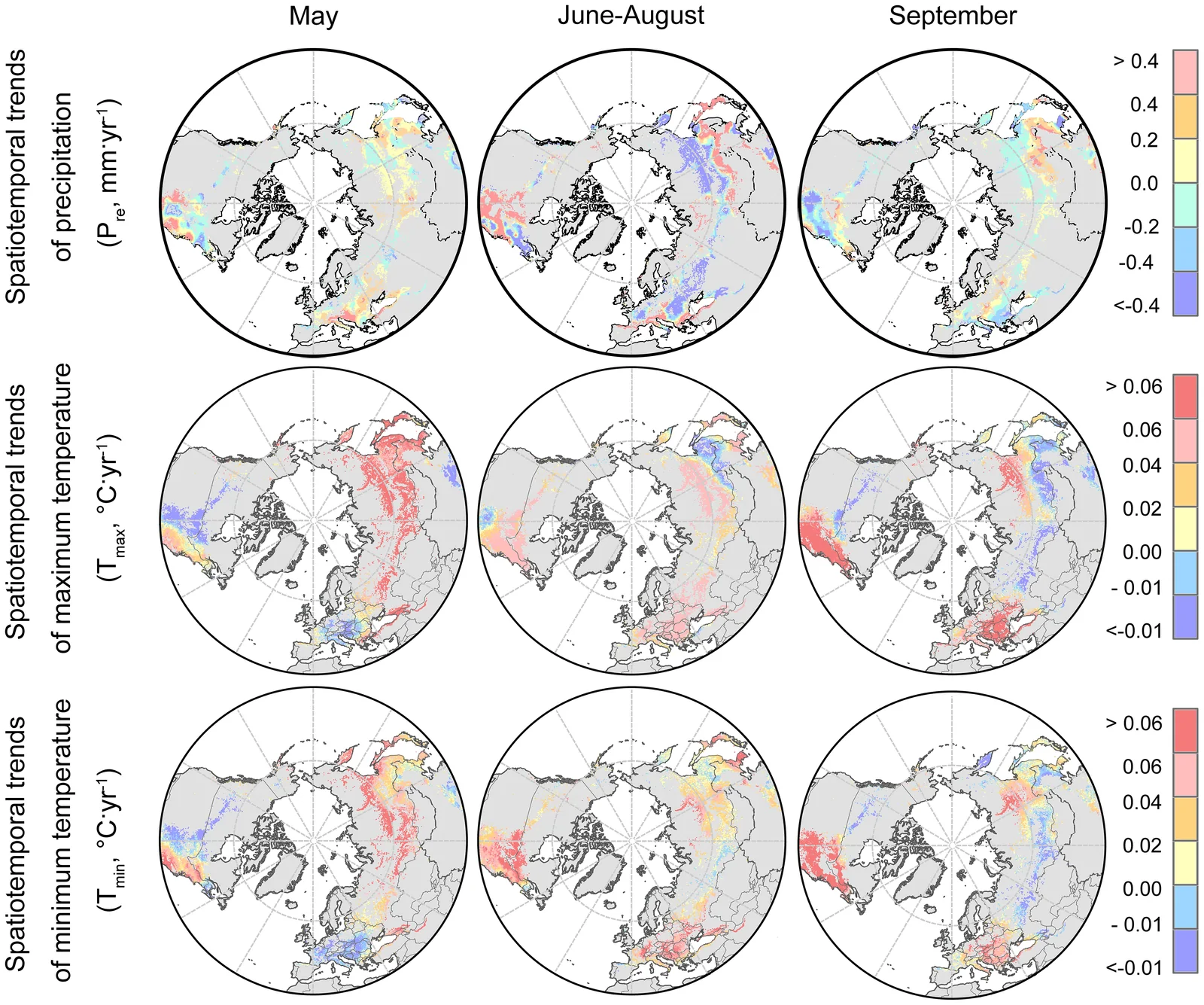
Spatiotemporal variations of meteorological factors (P_re_, T_max_, and T_min_) of intra-seasonal periods (May, June–August, and September) in the deciduous forest distribution area of the Northern Hemisphere from 2003 to 2021.

### 3.3 Relationships between LAI and meteorological factors

This study first analyzed the LAI_DF_–climate interactions across the growing season from 2003 to 2021. Our analysis showed that the LAI_DF_ showed a notable positive correlation with temperature, especially T_max_ (*p* = 0.002), whereas its relationship with precipitation was not significant (**Figure 6**). For both DBF and DNF, T_max_ was highly significantly positively related to LAI_DF_ (*p* < 0.01), indicating that T_max_ was strongly associated with an increase in LAI for different leaf forms. To assess the relative importance of these climatic factors, we further analyzed their independent contributions to the LAI dynamics. The results showed that the T_max_, T_min_, and P_re_ change approximately explained 52.0% of the LAI changes. In addition, three variables jointly explained 66.2% and 61.9% of the interannual LAI variance for DBF and DNF, respectively. Specifically, T_max_ was the predominant factor in both forest types, accounting for 43.1% and 37.0% of the explained variance, respectively—substantially more than the contributions of T_min_ and precipitation. In contrast, T_min_ contributed 18.5% in DBF versus 12.8% in DNF, whereas precipitation exhibited a reversed pattern, rising from 4.6% in DBF to 12.1% in DNF.

**Figure 6:**
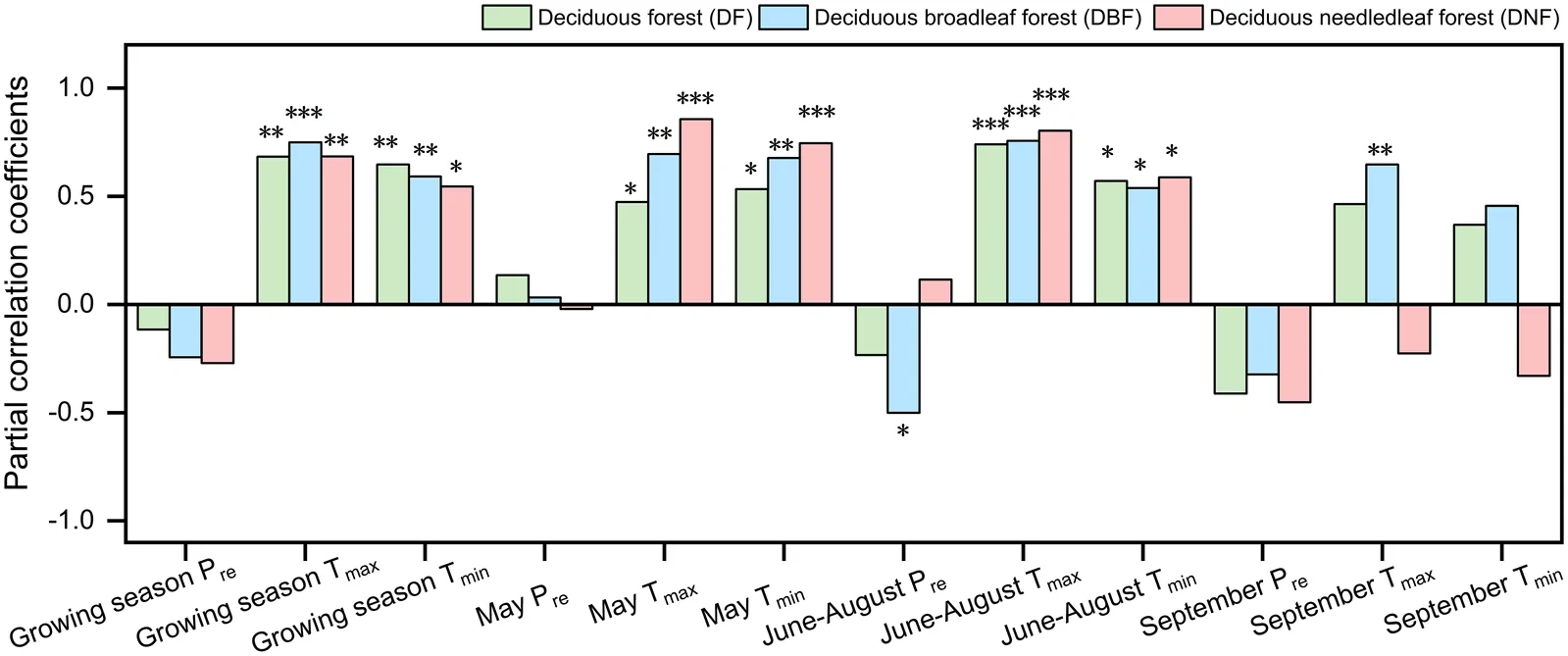
Partial correlation analyses between Leaf Area Index (LAI) of deciduous forests and meteorological factors over the growing season and its component periods. (*, **, *** represent *p* < 0.05, *p* < 0.01, and *p* < 0.001). Each group contains 19 annual sample observations.

We further found that LAI_DF_ (including LAI_DBF_ and LAI_DNF_) was significantly positively correlated with temperatures during May and June–August, and the association between temperature and LAI_DNF_ in May was more significant (*p* < 0.01, **Figure 6**). In September, the LAI_DBF_ presented significant positive dependences on both T_max_ (*p* = 0.004), in contrast, the LAI_DNF_ was not significantly correlated with temperatures (*p* = 0.368). In terms of the impacts of intra-seasonal P_re_ change on LAI_DF_, LAI_DF_ correlated negatively with precipitation across seasons, and the significant negative relationship between LAI_DBF_ and P_re_ was statistically significant during June–August.

Spatially, most regions exhibited a negative response to growing season P_re_ and LAI_DF_, with the most pronounced association in DNF of northeast China and Siberia (**Figures 7a, c**). Increased diurnal temperatures during the growing season may be related to the increase of LAI_DF_, but for some regions, the association of the diurnal warming with LAI_DF_ was asymmetric (**Figures 7b, c, e, f**). Specifically, rising T_max_ had a notably stronger positive effect on LAI_DF_ in most regions, whereas the response to nighttime warming showed spatial heterogeneity: the T_min_ exhibited a positive correlation with for most regions, while in northeast China, the relationship between LAI_DNF_ and the T_min_ had a negative correlation (**Figures 7b, c, e, f**).

**Figure 7:**
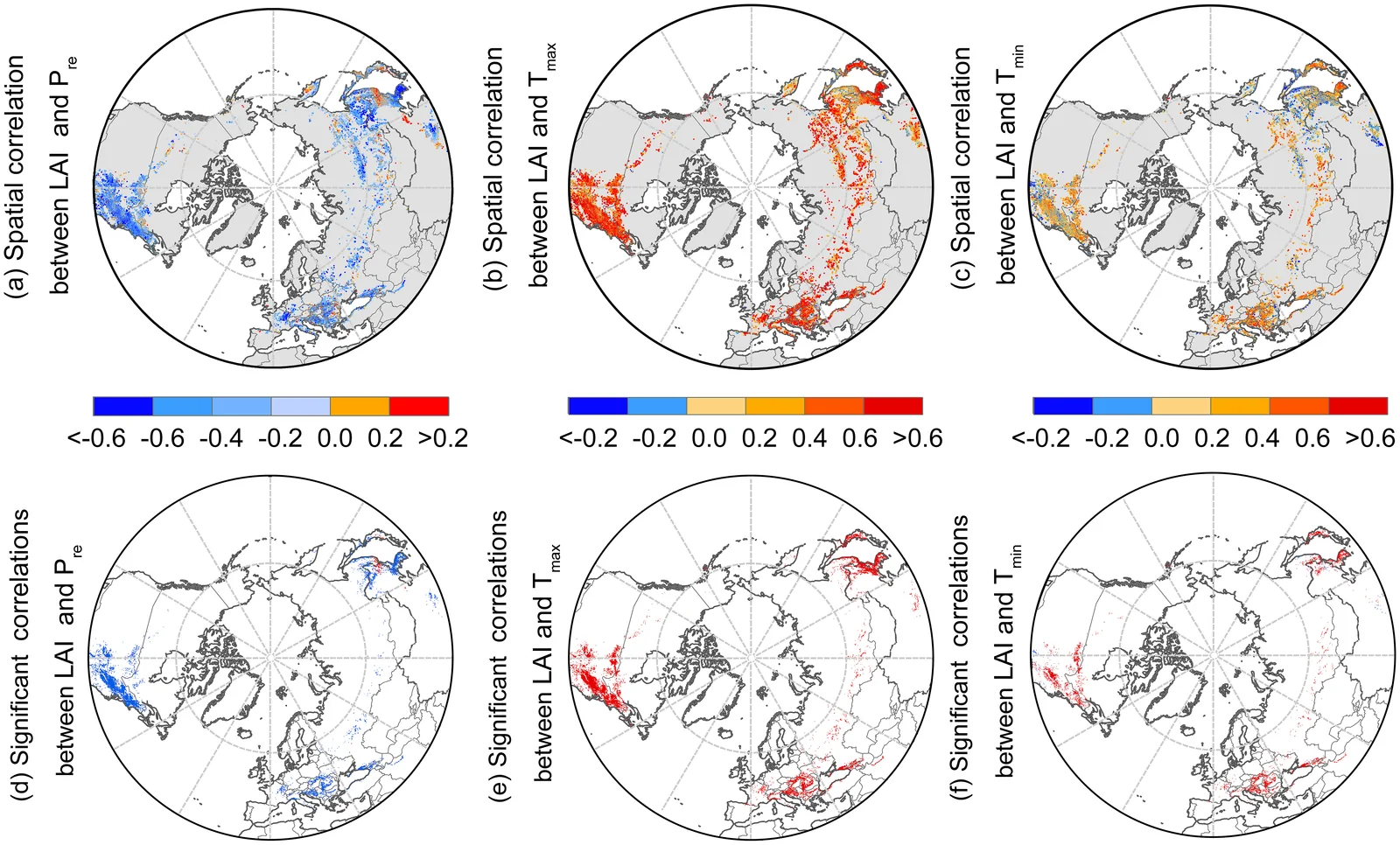
Spatial correlations between Leaf Area Index (LAI) of deciduous forests (deciduous needleleaf forests: approximately 7,745,951 pixels; deciduous broadleaf forests: approximately 8,463,342 pixels) and precipitation (P_re_), maximum (T_max_), and minimum temperature (T_min_) during the growing season in the Northern Hemisphere from 2003 to 2021. **(a)** Spatial correlation between LAI and Pre; **(b)** spatial correlation between LAI and Tmax; **(c)** spatial correlation between LAI and Tmin. **(d–f)** show significant positive (red) and negative (blue) correlations between LAI andclimatic factors based on FDR-adjusted significance (*q* = 0.05).

The spatial associations of intra-seasonal meteorological factors on LAI_DF_ showed that precipitation in different periods was usually negatively correlated with LAI_DF_ (**Figure 8**). The impact of temperatures on LAI_DF_ was positive, with a significant positive relationship between the T_max_ and LAI_DF_ during June–August, indicating that increasing temperatures were the key factor associated with the significant increase in LAI_DF_ for different leaf forms. In addition, we found that LAI_DF_ responded asymmetrically to daytime versus nighttime temperatures in northeast China and the eastern United States during mid-to-late growing season, and the asymmetric associations in these two regions had certain differences (**Figure 8**). The LAI_DF_ in northeast China was positively associated with T_max_ but negatively with T_min_ in that period; in the eastern United States, LAI_DF_ was positively linked to both T_max_ and T_min_ during those periods, but the positive effect of T_max_ was more significant.

**Figure 8:**
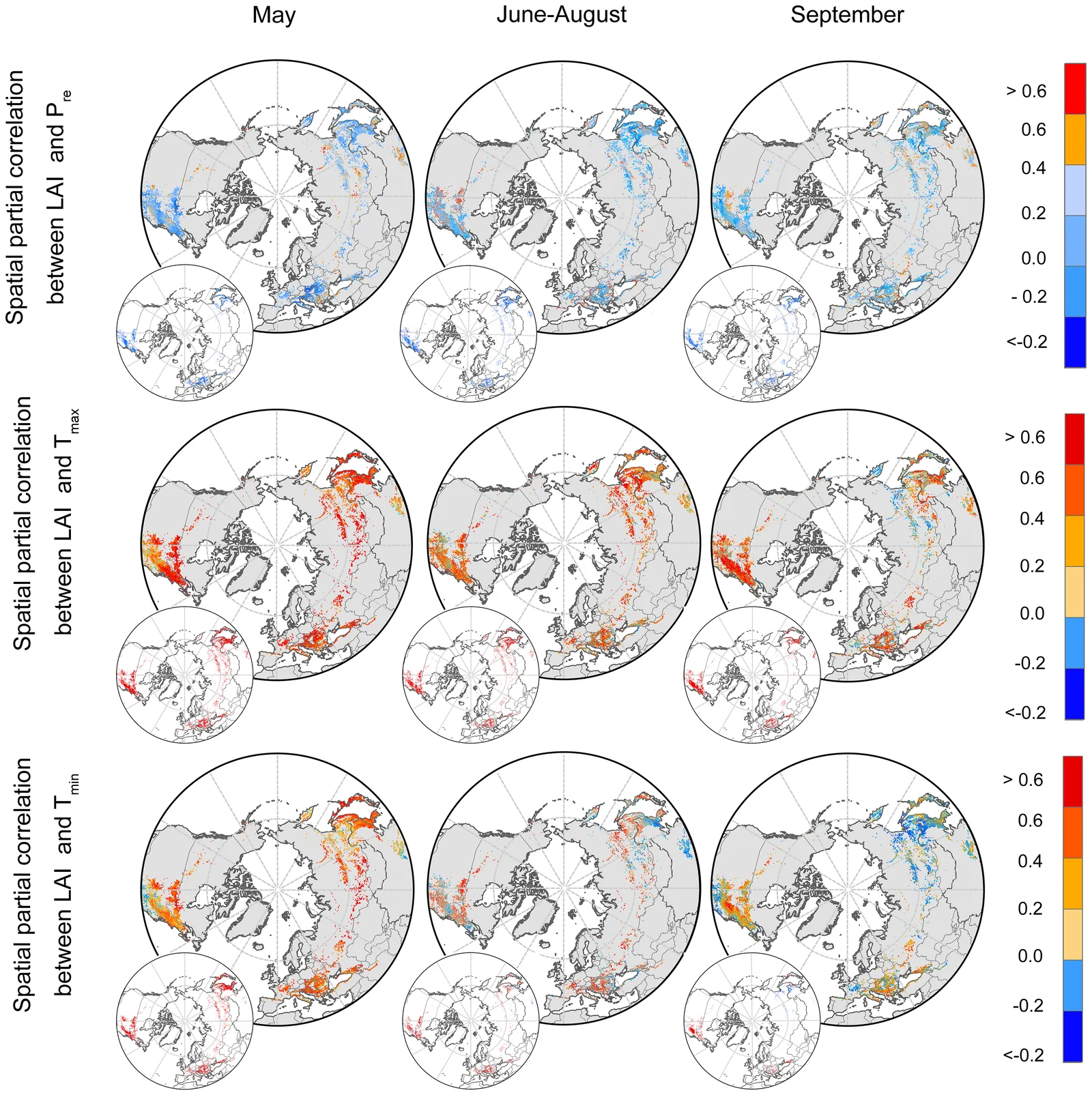
Spatial partial correlations between Leaf Area Index (LAI) of deciduous forests (deciduous needleleaf forests: approximately 7,745,951 pixels; deciduous broadleaf forests: approximately 8,463,342 pixels) and precipitation (P_re_), maximum (T_max_), and minimum temperature (T_min_) during the different periods in the Northern Hemisphere. The small inset maps show significant positive (red) and negative (blue) correlations between LAI and climatic factors based on FDR-adjusted significance (*q* = 0.05).

To further examine the response of deciduous forest LAI to climate variability, we quantified its sensitivity using the regression slope between LAI and meteorological variables (**Figure 9**), with the aggregated slopes representing the mean coefficients derived from the regression models. Sensitivity values of precipitation are almost entirely distributed on the negative side, verifying the overall negative LAI response to precipitation across all latitudinal zones of Northern Hemisphere deciduous forests. By contrast, sensitivity values of T_max_ and T_min_ shift from near zero at lower latitudes to strongly positive values approaching 0.02 at high latitudes, demonstrating a significant latitudinal increasing trend of temperature-driven LAI promotion effect. The spatial analysis results indicated that the sensitivity of LAI_DF_ to precipitation was negative, whereas its overall sensitivity to temperature was positive. Overall, the results of the linear regression analyses indicated that S_pre_, S_Tmax_, and S_Tmin_ were -0.01 m^2^m^-2^/mm, 0.16 m^2^m^-2^/°C, and 0.21 m^2^m^-2^/°C, respectively, indicating that from 2003 to 2021, for each additional 1 mm of precipitation during the growing season, LAI_DF_ decreased by 0.01 m^2^m^-2^. However, for every 1 °C increase in T_max_ and T_min_, the LAI_DF_ increased 0.16 m^2^m^-2^ and 0.21 m^2^m^-2^, respectively.

**Figure 9:**
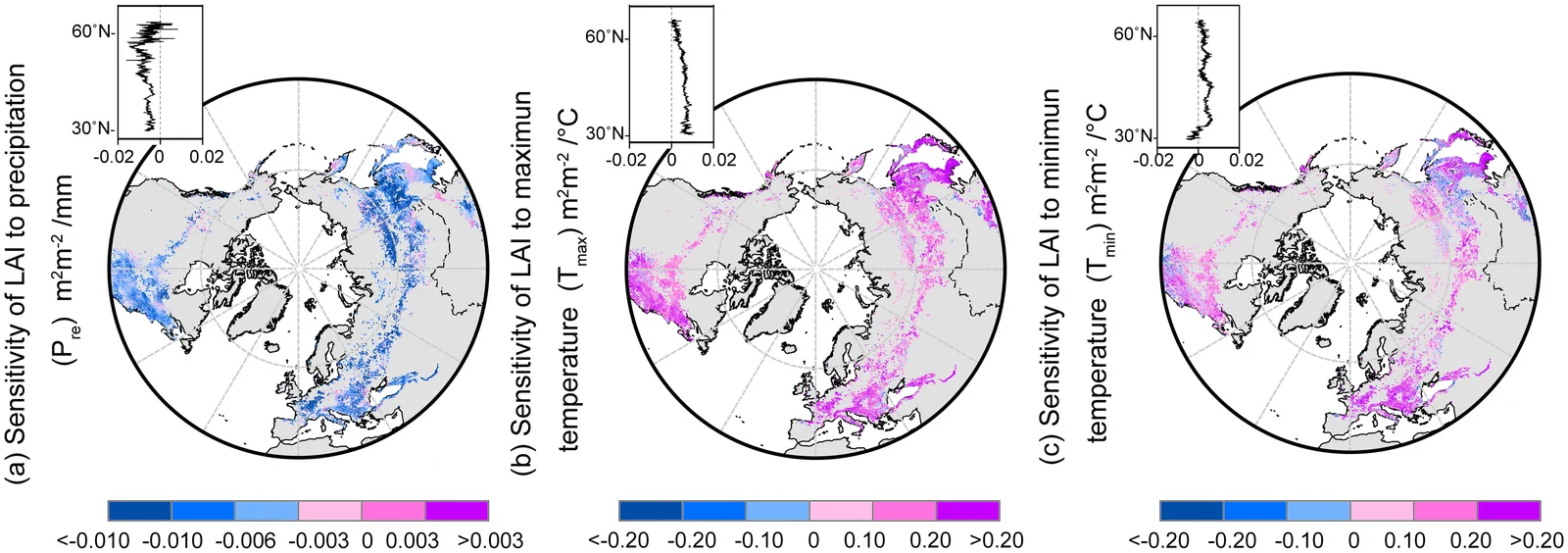
Spatial sensitivity of Leaf Area Index (LAI) of deciduous forests (deciduous needleleaf forests: approximately 7,745,951 pixels; deciduous broadleaf forests: approximately 8,463,342 pixels) to climate change during the growing season. **(a)** Sensitivity of deciduous forest LAI to precipitation (P_re_); **(b)** Sensitivity of deciduous forest LAI to maximum temperature (T_max_); **(c)** Sensitivity of deciduous forest LAI to minimum temperature (T_min_). The small inset plots of each panel are latitudinal mean values of LAI climate sensitivity; blue tones denote negative climatic sensitivity (climate factors suppress LAI), while orange tones represent positive climatic sensitivity (climate factors are consistent with LAI).

To formally test whether the observed differences in LAI sensitivity to daytime versus nighttime temperatures are statistically supported, we fitted T_max_ and T_min_ jointly in a multiple regression model and applied Wald tests to compare their coefficients. The partial regression coefficients (*β*) from the joint models showed that T_max_ was positively associated with LAI in both DBF (*β* = 0.329, 95% CI: 0.166 ~ 0.491, *p* < 0.001) and DNF (*β* = 0.250, 95% CI: 0.123 ~ 0.376, *p* < 0.001), whereas T_min_ showed a negative association in DBF (*β* = -0.185, 95% CI: -0.369 ~ -0.002, *p* = 0.048) and was not statistically significant in DNF (*β* = -0.170, 95% CI: -0.388 ~ 0.048, *p* = 0.118). Wald tests confirmed that the coefficients of T_max_ and T_min_ differed significantly for both DBF (F = 10.53, *p* = 0.005) and DNF (F = 7.33, *p* = 0.016), supporting the asymmetric diurnal sensitivity patterns described above. In addition, the apparent difference between the simple regression slopes and the joint model coefficients arises because T_max_ and T_min_ are moderately correlated (VIF = 6~8), meaning that the positive coefficient for T_min_ in the simple regression partly reflects its covariation with T_max_. The joint model, however, isolates the independent effect of each temperature variable after controlling for the other. Once the dominant daytime warming signal (T_max_) is accounted for, the residual effect of T_min_ becomes neutral or slightly negative. This interpretation is supported by the Wald test, which confirms that the two coefficients differ significantly.

To further validate our trend-based attribution and isolate interannual signals from long-term co-variation, we repeated the partial correlation analysis using first-differenced time series (n = 18 year-to-year changes) as a robustness check (**Supplementary Table S1**). In the differenced series, T_max_ remained significantly and positively associated with LAI for both DBF (*p* = 0.004) and DNF (*p* = 0.034). Interestingly, the negative association between T_min_ and LAI became statistically significant in DBF (*p* = 0.019), while remaining non-significant in DNF (*p* = 0.201; **Supplementary Table S1**). These results further validate our Wald test findings and underscore the contrasting effects of daytime versus nighttime warming on forest forests. Precipitation showed no significant partial correlation in the differenced series for any forest type (**Supplementary Table S1**), suggesting that temperature variability is the primary driver of interannual LAI fluctuations in these ecosystems.

## 4 Discussion

### 4.1 Growing season and seasonal variations of deciduous forest LAI

The multi-year average of growing season LAI_DF_ (3.45 m^2^m^-2^) in this study aligns with Liu et al. (2017) but shows lower values than their 3.0 ~ 5.0 m² m^-2^ LAI range. This divergence likely reflects our study’s focus on the LAI over the entire growing season, contrasting with their peak-season estimates from layered inversion. In addition, for high-latitude regions, the value of LAI_DF_ was low but increased significantly (**Figure 1**). Conversely, low-latitude regions exhibited high LAI but were subject to a declining trend of LAI_DF_. This latitudinal dichotomy corresponds to regional differences in topography and the spatial distribution of water and heat conditions, which are likely underlying factors (Ma et al., 2024). This study further found that the multi-year average values of LAI across different leaf forms all showed a pattern of being the lowest in May, followed by September, and the highest in June–August. This also verified previous findings that the vegetation coverage dynamics were the strongest in summer (Yang et al., 2017).

Overall, LAI_DF_ increased significantly during the growing season, at the rate of 0.81·10^–2^ m^2^m^-2^yr^-1^ from 2003 to 2021 (**Figure 3**). This is slightly lower than the interannual variation trend obtained in previous studies (approximately 1.00 ~ 2.00 10^–2^ m^2^m^-2^yr^-1^). The differences in the upward trend might be because our study period (2003–2021) differs from that of Ding et al. (2020), which spanned 1982–2020. In addition, although the LAI_DF_ across different leaf forms all showed significant increasing trends during the growing season, LAI_DNF_ only rose significantly during June–August (1.20·10^–2^ m^2^m^-2^yr^-1^), while LAI_DBF_ showed a significant upward trend in both June–August (0.75·10^–2^ m^2^m^-2^yr^-1^) and September (0.82·10^–2^ m^2^m^-2^yr^-1^, **Figure 3**). Findings from earlier studies suggest that the greening trend in the Northern Hemisphere was the most significant in summer, which is consistent with the optimal hydrothermal conditions and peak vegetation growth during this period. In this study, the seasonal increase patterns of LAI across different forms were highly consistent with this background (Zhu et al., 2016; Chen et al., 2019), which also confirms the sustained improvement of regional forest dynamics (Mao et al., 2012).

### 4.2 Impacts of hydrothermal changes on the LAI of deciduous forests

Our results suggest that increased precipitation was partially associated with the decreased LAI_DF_, whereas Hu et al. (2021) concluded it was the primary factor. This divergence likely stems from differences in the climatic backgrounds of the respective study areas. The research of Hu et al. (2021) mainly focused on arid regions, where water typically limits vegetation dynamics, and increased precipitation was positively associated with LAI by enhancing vegetation growth and leaf development. For the mid-high latitude regions that our study focuses on, increased rainfall is often accompanied by the increase in cloud cover, which in turn may limit photosynthesis and thus is associated with a negative LAI response (Graham et al., 2003). Intra-seasonally, increased precipitation of June–August exhibited a significantly negative relationship with LAI_DF_ (**Figures 6**, **8**). This may be because photosynthesis, a key factor of peak vegetation growth (Ma et al., 2024), is suppressed by reduced solar radiation during wetter summers (Ishibashi and Terashima, 1995; Chen et al., 2022).

For different forest types, LAI_DNF_ showed significant negative correlations with precipitation during the growing season and all seasons (**Figures 6**, **8**), suggesting that increased precipitation was associated with a decrease in LAI_DNF_. This is similar to the result previously pointed out by Liu et al. (2012), who report a significant negative link between P_re_ and LAI in the northern Greater Khingan Mountains. In these cold, high-latitude regions, precipitation does not appear to act as the primary constraint on forest dynamics. One possible explanation for the observed negative association is that increased rainfall may introduce multiple co-limiting factors. For instance, higher precipitation often accompanies reduced solar radiation and lower temperatures, which may suppress transpiration (Liu et al., 2012) and, together with seasonal waterlogging, create conditions that could potentially impede root respiration and vegetation growth (Zhang et al., 2002). Furthermore, increased precipitation can induce soil moisture saturation leading to anaerobic conditions, particularly in cold high-latitude forests such as those in Siberia and northeast China (Domisch et al., 2020). We emphasize that these physiological pathways are speculative and require direct experimental validation; however, consistent with existing hypotheses, such stress may suppress root respiratory activity, which could contribute to a reduction in LAI (Kreuzwieser and Rennenberg, 2014). These phenomena offer potential explanations for our results, although the underlying physiological mechanisms remain to be confirmed with more direct measurements in future investigations.

Conversely, LAI_DF_ was significantly positively correlated with growing season temperatures (**Figures 6**-**8**), suggesting that warming was positively associated with the leaf area index of deciduous forests across the Northern Hemisphere, albeit with notable heterogeneity across intra-seasonal and leaf forms. We found that warming temperatures during the growing season, particularly in the early stage, was the key factor associated with LAI_DF_ increases. In cold regions, warming in the early stage of the growing season shows a strong positive association with LAI, a pattern that is consistent with the hypothesis that warmer temperatures stimulate photosynthetic rates (Wu et al., 2012). This effect is more significant in deciduous needleleaf forests than in broadleaf forests (**Figure 6**), as temperature serves as the pivotal ecological factor in high-latitude cold zones during early growth. Based on established plant physiological theory, spring warming may advance leaf-onset and prolong the photosynthetic period, thereby increasing total carbon assimilation (Stagakis et al., 2022), which may contribute to the pronounced increase in LAI_DF_ (**Figures 1**-**6**).

Interestingly, leaf form had a stronger influence on the LAI_DF_–climate relationship in autumn than in other seasons (**Figures 6**, **8**). Specifically, increased diurnal temperatures during September showed a significantly associated with LAI_DBF_, but not with LAI_DNF_. This divergence is consistent with fundamental physiological and ecological differences in the survival strategies. Deciduous broadleaf forests employ an ‘opportunistic’ strategy, characterized by high-cost, high-return leaves (Flo et al., 2021), which are thought to enable them to capitalize on any favorable conditions to prolong photosynthesis (Bond, 1989; Ma et al., 2023). Consistent with established plant physiological theory, late-stage warming may be associated with delayed senescence, prolonging photosynthesis and ultimately contributing to a significant increase in LAI_DBF_ (**Figures 1**, **6**, **8**). In contrast, deciduous needleleaf forests adopt a ‘conservative’ strategy (Ma et al., 2023), display structural leaf traits (e.g., thick cuticles, low nitrogen concentrations, stomatal conductance, or leaf nitrogen concentrations) that, according to previous studies, have been hypothesized to reduce transpiration demand and potentially lower sensitivity to moisture conditions at the late growing season (Bond, 1989; Flo et al., 2021). Meanwhile, increased precipitation in that final month may suppress LAI_DNF_ by elevating air humidity and reducing photosynthetically active radiation, thereby limiting photosynthesis (Chen et al., 2022). Therefore, LAI_DNF_ was insensitive to precipitation and temperature changes during that month.

Furthermore, the differential responses of LAI_DNF_ and LAI_DBF_ to September warming are associated not only with temperature but also with their intrinsic divergence in photoperiod sensitivity. Previous studies have established photoperiod as a potential signal regulating autumn phenology in temperate and boreal trees (Ekberg et al., 1979; Körner and Basler, 2010). Their survival strategies often prioritize photoperiod signals to ensure safe dormancy rather than pursuing carbon gain at the end of the season (Basler and Körner, 2014). Consequently, this further contributed to the differences in the responses of needleleaf and broadleaf forests to warming at that time.

As Peng et al. (2013) have pointed out, daytime and nighttime temperatures affect plant dynamics in the Northern Hemisphere asymmetrically. This study also found a distinct asymmetric pattern in the response of LAI_DF_ to diurnal warming during the growing season, with T_max_ exerting a markedly stronger positive association with LAI_DF_ (both in LAI_DBF_ and LAI_DNF_ for most regions) than T_min_ from June to September (**Figures 6**-**9**), indicating that T_max_ was strongly associated with increased LAI_DF_. This aligns with previous findings that vegetation is most strongly determined by daytime temperature changes (Wu et al., 2024). On one hand, the promotion of photosynthesis by rising daytime temperature mainly stems from its promotion of RuBisCO and other key photosynthetic enzymes (Cao et al., 2025): daytime warming effectively alleviates the limitations in the biochemical pathways of photosynthesis by enhancing the catalytic efficiency of enzymatic limits in leaves (Moore et al., 2021; Cao et al., 2025), which showed a positive association with the leaf activities and the extension of the growing season. Meanwhile, daytime warming may also be associated with accelerated nutrient cycling, as suggested by previous studies, thus further supporting the increase of LAI_DF_. On the other hand, rising T_min_ may exert a positive effect on LAI by reducing frost, or it may have a negative association with LAI by increasing respiration.

This study further found that increasing T_min_ had a complex impact on LAI_DBF_. For instance, in the eastern region of the United States, a region with favorable moisture and temperature conditions, LAI_DBF_ showed an asymmetric response to the diurnal temperature: LAI_DBF_ positively responded to T_max_ but not to T_min_, at a significant level (**Figure 7**). However, in high-latitude deciduous forests such as those in northeast China, increased T_max_ during May and September showed a significant positive association with LAI_DBF_, while increased T_min_ was associated with a decreasing trend of LAI_DBF_ (**Figures 1**, **7**, **8**). This phenomenon may be related to enhanced respiration from nighttime warming, which in turn may be associated with increased consumption of organic matter (Ma et al., 2022). However, under sufficient water and nutrient supply, vegetation may achieve net organic matter accumulation through the overcompensation effect: after nighttime respiration consumption, photosynthetic capacity increases the next day (Peng et al., 2013), suggesting that nighttime warming is beneficial for LAI_DBF_ in favorable temperature and precipitation conditions.

### 4.3 Explanation of changes in deciduous forests LAI across the Northern Hemisphere

As revealed by the correlations between the LAI_DF_ and climate factors, we conclude that increasing T_max_ and T_min_ during 2003–2021 were positively associated with the increasing LAI_DF_. For different leaf forms, increased LAI_DBF_ and LAI_DNF_ during May and June–August were also associated with temperature rises. Although leaf form exerts only a modest influence on LAI–climate interactions during the growing season, its role strengthens noticeably in September (**Figures 6**, **8**). Specifically, while precipitation showed a negative but not significant correlation with LAI_DBF_ of September, this was offset by the significant positive link to temperatures. Therefore, the significant warming in the deciduous broadleaf forest region during the study period might contribute to the increase in LAI_DBF_ in that final month. In contrast, LAI_DNF_ exhibited no significant correlation with temperature or precipitation during that month (**Figures 6**, **8**). From 2003 to 2021, the combination of warming and decreased precipitation might have jointly contributed to no statistically significant change in LAI_DNF_ in September, which further exacerbated the differences in LAI_DF_ during the same period.

Spatially, the LAI_DF_ increase in high-latitude regions, especially within deciduous needleleaf forests (**Figure 1**), is primarily associated with a warmer growing season, with consistent temperature increases in all seasons, as well as reduced precipitation during June–August (**Figures 4**, **5**). In contrast, the substantial increase in precipitation over the growing season and its component phases, in parts of the deciduous broadleaf forests of the eastern United States, might be related to the decrease in LAI_DF_ in this area. Global warming exhibits diurnal temperature asymmetry in many regions, with T_min_ generally increasing more rapidly than T_max_ (Davy et al., 2017). This study found that LAI_DF_ shows a stronger association with T_max_, while its correlation with T_min_ exhibited significant spatial variation (**Figures 7**, **8**). Although T_max_ increased slightly faster than T_min_ in deciduous forest regions (**Figures 4**, **5**), driving the overall rise in LAI_DF_, we highlight that sustained rapid nighttime warming in the future may weaken the canopy dynamics of high-latitude forests.

### 4.4 Limitations

It should be pointed out that this study may have several limitations. First, uncertainties in MODIS LAI and land-cover products arise from mixed pixels, classification errors, cloud/snow contamination, saturation, and missing values. Resampling the climate data to 500 m introduces spatial mismatches, and the lack of ground validation further constrains accuracy. Second, the 19-year time series is relatively short for robust trend attribution. Temporal and spatial autocorrelation among pixels reduce effective degrees of freedom, although we used HAC standard errors and based all formal inferences on aggregated series (n = 19) rather than pixel counts to mitigate this. The fixed May–September growing season window, validated by MCD12Q2 phenology, remains an approximation of actual SOS/EOS across latitudes. Notably, while MCD12Q2 supports this broad window, our intra-seasonal division into May, June–August, and September relies on fixed calendar bins rather than pixel-specific phenophases; this approach was deliberately adopted to ensure temporally synchronous comparisons between DBF and DNF, which exhibit fundamentally distinct phenological rhythms, although it inevitably overlooks finer-scale local phenological variations. Moderate collinearity between T_max_ and T_min_ complicates isolation of their individual effects, although partial correlation analysis partially addresses this issue. Most importantly, partial correlation analysis reveals statistical associations rather than causal mechanisms; future studies integrating process-based models or manipulative experiments could help move beyond correlative inference toward mechanistic understanding. Third, our climate-only analysis omitted several other potential drivers, including radiation, VPD, soil moisture, drought, CO_2_ fertilization, nitrogen deposition, nutrient limitations, and various disturbances (e.g., fire, insects, storms, harvest, and forest aging), nor did it account for lagged climatic effects and extreme events. Additionally, the marked geographic segregation of DBF and DNF means that leaf-form-associated differences inherently include environmental context. Future work integrating stratified climate-zone analyses, paired sampling designs, higher-resolution remote sensing, *in situ* measurements, disturbance records, and alternative detrending strategies, along with process-based models or manipulative experiments, would help move beyond correlative inference toward mechanistic understanding and better separate leaf-form effects from environmental covariation.

## 5 Conclusions

From 2003 to 2021, the LAI of deciduous forests during the growing season significantly increased by 0.81·10^–2^ m^2^m^-2^yr^-1^ across the Northern Hemisphere. Although LAI_DF_ generally showed increasing trends in the growing season, their intra-seasonal variation patterns were different: LAI_DBF_ showed significant increasing trends during both June–August and September, whereas the increase of LAI_DNF_ was mainly concentrated in June–August. For both deciduous broadleaf and needleleaf forests, precipitation showed a negative association with LAI across the growing season, while temperatures exhibited stronger positive associations with LAI in May and June–August. During the later stage of the growing season, however, warming temperatures generally associated with an increased LAI_DBF_ but showed no significant change in LAI_DNF_. We found asymmetric responses of LAI to daytime versus nighttime warming: daytime warming showed a larger positive association with LAI_DBF_ and LAI_DNF_ in the majority of regions. Our findings highlight the inter-seasonal distinct responses of deciduous broadleaf and needleleaf forests’ LAI to climate change and suggest that the asymmetric effects of diurnal warming on deciduous forests should be incorporated into forest canopy dynamic prediction models.

## Data Availability

The original contributions presented in the study are included in the article/**Supplementary Material**. Further inquiries can be directed to the corresponding authors.

## References

[B1] Asner G. P. Scurlock J. M. O. Hicke A. (2003). Global synthesis of leaf area index observations: implications for ecological and remote sensing studies. Global Ecol. Biogeogr. 12, 191–205. doi: 10.1046/j.1466-822X.2003.00026.x37945311

[B2] Axelrod D. I. (1966). Origin of deciduous and evergreen habits in temperate forests. Evolution 20, 1–15. doi: 10.2307/2406145 28564755

[B3] Basler D. Körner C. (2014). Photoperiod and temperature responses of bud swelling and bud burst in four temperate forest tree species. Tree Physiol. 34, 377–388. doi: 10.1093/treephys/tpu021 24713858

[B4] Becker P. (2000). Competition in the regeneration niche between conifers and angiosperms: Bond's slow seedling hypothesis. Funct. Ecol. 14, 401–412. doi: 10.1046/j.1365-2435.2000.00455.x

[B5] Bond W. J. (1989). The tortoise and the hare: ecology of angiosperm dominance and gymnosperm persistence. Biol. J. Linn. Soc 36, 227–249. doi: 10.1111/j.1095-8312.1989.tb00492.x40046247

[B6] Cao R. Zhang Y. Fernández-Martínez M. Zhang Z. Lai G. Ju W. . (2025). Global evidence for a positive relationship between tree species richness and ecosystem photosynthesis. Nat. Plants. 11, 1429–1440. doi: 10.1038/s41477-025-02046-1 40610738

[B7] Chen C. Park T. Wang X. Piao S. Xu B. Chaturvedi R. K. . (2019). China and India lead in greening of the world through land-use management. Nat. Sustain. 2, 122–129. doi: 10.1038/s41893-019-0220-7 30778399 PMC6376198

[B8] Chen R. Liu L. Liu X. (2022). The negative impact of excessive moisture contributes to the seasonal dynamics of photosynthesis in Amazon moist forests. Earths Future 10, e2021EF002306. doi: 10.1029/2021EF00230629143083

[B9] Cox D. T. C. Maclean I. M. D. Gardner A. S. Gaston K. J. (2020). Global variation in diurnal asymmetry in temperature, cloud cover, specific humidity and precipitation and its association with leaf area index. Glob. Change Biol. 26, 7099–7111. doi: 10.1111/gcb.15336 32998181

[B10] D’Orangeville L. Houle D. Duchesne L. Phillips R. P. Bergeron Y. Kneeshaw D. (2018). Beneficial effects of climate warming on boreal tree growth may be transitory. Nat. Commun. 9, 3213. doi: 10.1038/s41467-018-05705-4 30097584 PMC6086880

[B11] Davy R. Esau I. Chernokulsky A. Outten S. Zilitinkevich S. (2017). Diurnal asymmetry to the observed global warming. Int. J. Climatol. 37, 79–93. doi: 10.1002/joc.468842587375

[B12] Deng K. Henderson M. Liu B. Huang W. Chen M. Zheng P. . (2025). Cumulative and lagged effects of drought on the phenology of different vegetation types in East Asia 2001–2020. Remote Sensing-basel. 17, 2700. doi: 10.3390/rs1715270030654563

[B13] Deng G. Zhang H. Guo X. Shan Y. Ying H. Rihan W. . (2019). Asymmetric effects of daytime and nighttime warming on boreal forest spring phenology. Remote Sensing-basel. 11, 1651. doi: 10.3390/rs1114165130654563

[B14] Ding Z. Peng J. Qiu S. Zhao Y. (2020). Nearly half of global vegetated area experienced inconsistent vegetation growth in terms of greenness, cover, and productivity. Earths Future 8, e2020EF001618. doi: 10.1029/2020EF00161829143083

[B15] Domisch T. Qian J. Sondej I. Martz F. Lehto T. Piirainen S. . (2020). Here comes the flood! Stress effects of continuous and interval waterlogging periods during the growing season on Scots pine saplings. Tree Physiol. 40, 869–885. doi: 10.1093/treephys/tpaa036 32186742

[B16] Ekberg I. Eriksson G. Dormling I. (1979). Photoperiodic reactions in conifer species. Ecography 2, 255–263. doi: 10.1111/j.1600-0587.1979.tb01297.x40046247

[B17] Fang H. Baret F. Plummer S. Schaepman‐Strub G. (2019). An overview of global leaf area index (LAI): Methods, products, validation, and applications. Rev. Geophys. 57, 739–799. doi: 10.1029/2018RG00060829143083

[B18] Flo V. Martínez‐Vilalta J. Mencuccini M. Granda V. Anderegg W. R. Poyatos R. (2021). Climate and functional traits jointly mediate tree water‐use strategies. New Phytol. 231, 617–630. doi: 10.1111/nph.17404 33893652

[B19] Forzieri G. Dakos V. McDowell N. G. Ramdane A. Cescatti A. (2022). Emerging signals of declining forest resilience under climate change. Nature 608, 534–539. doi: 10.1038/s41586-022-04959-9 35831499 PMC9385496

[B20] Fu C. Qin S. Cheng L. Li L. Zhang Q. Lei X. . (2025). Estimating evapotranspiration from deciduous broadleaf forests by considering the effect of foliar litter. Agr. For. Meteorol. 373, 110736. doi: 10.1016/j.agrformet.2025.11073642574925

[B21] Fu Y. H. Zhao H. Piao S. Peaucelle M. Peng S. Zhou G. . (2015). Declining global warming effects on the phenology of spring leaf unfolding. Nature 526, 104–107. doi: 10.1038/nature15402 26416746

[B22] Graham E. A. Mulkey S. S. Kitajima K. Phillips N. G. Wright S. J. (2003. Cloud cover limits net CO2 uptake and growth of a rainforest tree during tropical rainy seasons. Proc. Natl. Acad. Sci. U.S.A. 100, 572–576. doi: 10.1073/pnas.0133045100 12518044 PMC141037

[B23] Gu H. Qiao Y. Xi Z. Rossi S. Smith N. G. Liu J. . (2022). Warming-induced increase in carbon uptake is linked to earlier spring phenology in temperate and boreal forests. Nat. Commun. 13, 3698. doi: 10.1038/s41467-022-31496-w 35760820 PMC9237039

[B24] Han H. Yin Y. Zhao Y. Qin F. (2023). Spatiotemporal variations in fractional vegetation cover and their responses to climatic changes on the Qinghai–Tibet plateau. Remote Sens-basel. 15, 2662. doi: 10.3390/rs1510266230654563

[B25] Hou M. Venäläinen A. K. Wang L. Pirinen P. Gao Y. Jin S. . (2020). Spatio-temporal divergence in the responses of Finland’s boreal forests to climate variables. Int. J. Appl. Earth Obs. 92, 102186. doi: 10.1016/j.jag.2020.10218642574925

[B26] Hu Y. Li H. Wu D. Chen W. Zhao X. Hou M. . (2021). LAI-indicated vegetation dynamic in ecologically fragile region: A case study in the Three-North Shelter Forest program region of China. Ecol. Indic. 120, 106932. doi: 10.1016/j.ecolind.2020.10693242574925

[B27] Ishibashi M. Terashima I. (1995). Effects of continuous leaf wetness on photosynthesis: adverse aspects of rainfall. Plant Cell Environ. 18, 431–438. doi: 10.1111/j.1365-3040.1995.tb00377.x40046247

[B28] Jiang C. Yeddula S. D. Ku W. S. (2024). A convolutional neural network model for accurate short-term leaf area index prediction. Model. Earth Syst. Env. 10, 6397–6412. doi: 10.1007/s40808-024-02090-430311153

[B29] Kang Y. Özdoğan M. Zipper S. C. Román M. O. Walker J. Hong S. Y. . (2016). How universal is the relationship between remotely sensed vegetation indices and crop leaf area index? A global assessment. Remote Sensing-basel. 8, 597. doi: 10.3390/rs8070597 30002923 PMC6038712

[B30] Kikuzawa K. (1991). A cost-benefit analysis of leaf habit and leaf longevity of trees and their geographical pattern. Am. Nat. 138, 1250–1263. doi: 10.1086/285280 33442259 PMC7797338

[B31] Körner C. Basler D. (2010). Phenology under global warming. Science 327, 1461–1462. doi: 10.1126/science.1186473 20299580

[B32] Kreuzwieser J. Rennenberg H. (2014). Molecular and physiological responses of trees to waterlogging stress. Plant Cell Environ. 37, 2245–2259. doi: 10.1111/pce.12310 24611781

[B33] Lee L. X. Whitby T. G. Munger J. W. Stonebrook S. J. Friedl M. A. (2023). Remote sensing of seasonal variation of LAI and fAPAR in a deciduous broadleaf forest. Agr. For. Meteorol. 333, 109389. doi: 10.1016/j.agrformet.2023.10938942574925

[B34] Li Z. Fang H. Tu J. Li X. Sha Z. (2022). Phenological shifts of the deciduous forests and their responses to climate variations in North America. Forests 13, 1137. doi: 10.3390/f1307113730654563

[B35] Li W. Manzanedo R. D. Jiang Y. Ma W. Du E. Zhao S. . (2023). Reassessment of growth-climate relations indicates the potential for decline across Eurasian boreal larch forests. Nat. Commun. 14, 3358. doi: 10.1038/s41467-023-39057-5 37291110 PMC10250375

[B36] Liu Y. Ju W. Chen J. Zhu G. Xing B. Zhu J. . (2012). Spatial and temporal variations of forest LAI in China during 2000–2010. Chin. Sci. Bull. 57, 2846–2856. doi: 10.1007/s11434-012-5064-830311153

[B37] Liu Y. Liu R. Pisek J. Chen J. M. (2017). Separating overstory and understory leaf area indices for global needleleaf and deciduous broadleaf forests by fusion of MODIS and MISR data. Biogeosciences 14, 1093–1110. doi: 10.5194/bg-14-1093-2017

[B38] Liu Y. Wu C. Wang X. Zhang Y. (2023). Contrasting responses of peak vegetation growth to asymmetric warming: Evidences from FLUXNET and satellite observations. Glob. Change Biol. 29, 2363–2379. doi: 10.1111/gcb.16592 36695551

[B39] Lv J. Zhao W. He B. Jiang G. (2024). Asymmetric warming has different impacts on vegetation growth across various vegetation types on the Tibetan Plateau. GISci. Remote Sens. 61, 2348257. doi: 10.1080/15481603.2024.234825737339054

[B40] Ma H. Crowther T. W. Mo L. Maynard D. S. Renner S. S. Van den Hoogen J. . (2023). The global biogeography of tree leaf form and habit. Nat. Plants 9, 1795–1809. doi: 10.1038/s41477-023-01543-5 37872262 PMC10654052

[B41] Ma R. Shen X. Zhang J. Xia C. Liu Y. Wu L. . (2022). Variation of vegetation autumn phenology and its climatic drivers in temperate grasslands of China. Int. J. Appl. Earth Obs. 114, 103064. doi: 10.1016/j.jag.2022.10306442574925

[B42] Ma Y. Wang W. Jin S. Li H. Liu B. Gong W. . (2023). Spatiotemporal variation of LAI in different vegetation types and its response to climate change in China from 2001 to 2020. Ecol. Indic. 156, 111101. doi: 10.1016/j.ecolind.2023.11110142574925

[B43] Ma R. Zhang J. Shen X. Liu B. Lu X. Jiang M. (2024). Impacts of climate change on fractional vegetation coverage of temperate grasslands in China from 1982 to 2015. J. Environ. Manage. 350, 119694. doi: 10.1016/j.jenvman.2023.119694 38035505

[B44] Mao J. Shi X. Thornton P. E. Piao S. Wang X. (2012). Causes of spring vegetation growth trends in the northern mid–high latitudes from 1982 to 2004. Environ. Res. Lett. 7, 14010. doi: 10.1088/1748-9326/7/1/014010

[B45] Mo L. Zohner C. M. Reich P. B. Liang J. De Miguel S. Nabuurs G. J. . (2023). Integrated global assessment of the natural forest carbon potential. Nature 624, 92–101. doi: 10.1038/s41586-023-06723-z 37957399 PMC10700142

[B46] Moore C. E. Meacham-Hensold K. Lemonnier P. Slattery R. A. Benjamin C. Bernacchi C. J. . (2021). The effect of increasing temperature on crop photosynthesis: from enzymes to ecosystems. J. Exp. Bot. 72, 2822–2844. doi: 10.1093/jxb/erab090 33619527 PMC8023210

[B47] Munier S. Carrer D. Planque C. Camacho F. Albergel C. Calvet J. C. (2018). Satellite leaf area index: Global scale analysis of the tendencies per vegetation type over the last 17 years. Remote Sensing-basel. 10, 424. doi: 10.3390/rs1003042430654563

[B48] O’brien R. M. (2007). A caution regarding rules of thumb for variance inflation factors. Qual. Quant. 41, 673–690. doi: 10.1007/s11135-006-9018-630311153

[B49] Pan Y. Birdsey R. A. Phillips O. L. Houghton R. A. Fang J. Kauppi P. E. . (2024). The enduring world forest carbon sink. Nature 631, 563–569. doi: 10.1038/s41586-024-07602-x 39020035

[B50] Park H. Jeong S. (2021). Leaf area index in Earth system models: how the key variable of vegetation seasonality works in climate projections. Environ. Res. Lett. 16, 034027. doi: 10.1088/1748-9326/abe2cf

[B51] Peng S. Piao S. Ciais P. Myneni R. B. Chen A. Chevallier F. . (2013). Asymmetric effects of daytime and night-time warming on Northern Hemisphere vegetation. Nature 501, 88–92. doi: 10.1038/nature12434 24005415

[B52] Piao S. Fang J. Zhou L. Ciais P. Zhu B. (2006). Variations in satellite‐derived phenology in China's temperate vegetation. Glob. Change Biol. 12, 672–685. doi: 10.1111/j.1365-2486.2006.01123.x40046247

[B53] Piao S. Wang X. Ciais P. Zhu B. Wang T. A. O. Liu J. I. E. (2011). Changes in satellite‐derived vegetation growth trend in temperate and boreal Eurasia from 1982 to 2006. Glob. Change Biol. 17, 3228–3239. doi: 10.1111/j.1365-2486.2011.02419.x40046247

[B54] Piao S. Wang X. Park T. Chen C. Lian X. He Y. . (2020). Characteristics, drivers and feedbacks of global greening. Nat. Rev. Earth Env. 1, 14–27. doi: 10.1038/s43017-019-0001-x37880705

[B55] Shen X. Liu Y. Zhang J. Wang Y. Ma R. Liu B. . (2022). Asymmetric impacts of diurnal warming on vegetation carbon sequestration of marshes in the Qinghai Tibet Plateau. Global Biogeochem. Cy 36, e2022GB007396. doi: 10.1029/2022GB00739629143083

[B56] Shen X. Shen M. Wu C. Peñuelas J. Ciais P. Zhang J. . (2024). Critical role of water conditions in the responses of autumn phenology of marsh wetlands to climate change on the Tibetan Plateau. Glob. Change Biol. 30, e17097. doi: 10.1111/gcb.17097 38273510

[B57] Shi H. Zhou Q. He R. Zhang Q. Dang H. (2023). Asymmetric effects of daytime and nighttime warming on alpine treeline recruitment. Glob. Change Biol. 29, 3463–3475. doi: 10.1111/gcb.16675 36897639

[B58] Stagakis S. Markos N. Vanikiotis T. Levizou E. Kyparissis A. (2022). Multi-year monitoring of deciduous forests ecophysiology and the role of temperature and precipitation as controlling factors. Plants 11, 2257. doi: 10.3390/plants11172257 36079636 PMC9460110

[B59] Tan J. Piao S. Chen A. Zeng Z. Ciais P. Janssens I. A. . (2015). Seasonally different response of photosynthetic activity to daytime and night‐time warming in the Northern Hemisphere. Glob. Change Biol. 21, 377–387. doi: 10.1111/gcb.12724 25163596

[B60] Tang Y. Du E. Xue B. Peñuelas J. Reich P. B. (2026). Reversibility of interannual tree growth variability in Southern Boreal Forest. Glob. Change Biol. 32, e70696. doi: 10.1111/gcb.70696 41542714

[B61] Tao F. Chen Y. Fu B. (2020). Impacts of climate and vegetation leaf area index changes on global terrestrial water storage from 2002 to 2016. Sci. Total Environ. 724, 138298. doi: 10.1016/j.scitotenv.2020.138298 32272410

[B62] Tao W. Mao K. He J. Smith N. G. Qiao Y. Guo J. . (2022). Daytime warming triggers tree growth decline in the Northern Hemisphere. Glob. Change Biol. 28, 4832–4844. doi: 10.1111/gcb.16238 35561010

[B63] Wang D. Ziegler A. D. Holden J. Spracklen D. V. Ciais P. Peng L. . (2025). Vegetation cover change as a growing driver of global leaf area index dynamics. Nat. Commun. 16, 9259. doi: 10.1038/s41467-025-64305-1 41115865 PMC12537924

[B64] Wu S. H. Jansson P. E. Kolari P. (2012). The role of air and soil temperature in the seasonality of photosynthesis and transpiration in a boreal Scots pine ecosystem. Agr. For. Meteorol. 156, 85–103. doi: 10.1016/j.agrformet.2012.01.00642574925

[B65] Wu X. Liu H. Li X. Liang E. Beck P. S. Huang Y. (2016). Seasonal divergence in the interannual responses of Northern Hemisphere vegetation activity to variations in diurnal climate. Sci. Rep. 6, 19000. doi: 10.1038/srep19000 26751166 PMC4707447

[B66] Wu L. Shen X. Lu X. Jiang M. (2024). Asymmetric effects of diurnal warming on carbon allocation to leaves of marsh wetlands on the Tibetan Plateau. Int. J. Digit. Earth 17, 2419936. doi: 10.1080/17538947.2024.241993637339054

[B67] Wu D. Zhao X. Liang S. Zhou T. Huang K. Tang B. . (2015). Time‐lag effects of global vegetation responses to climate change. Glob. Change Biol. 21, 3520–3531. doi: 10.1029/2020JG005698 25858027

[B68] Xue B. L. Guo Q. Gong Y. Hu T. Liu J. Ohta T. (2016). The influence of meteorology and phenology on net ecosystem exchange in an eastern Siberian boreal larch forest. J. Plant Ecol. 9, 520–530. doi: 10.1093/jpe/rtv075

[B69] Yang B. Chen L. Li S. Yi B. Hu N. (2025). Revealing long-term runoff mechanisms using the deep learning model interpretable framework driven by global and local information. J. Hydrol. 663, 134167. doi: 10.1016/j.jhydrol.2025.13416742574925

[B70] Yang H. Yang X. Heskel M. Sun S. Tang J. (2017). Seasonal variations of leaf and canopy properties tracked by ground-based NDVI imagery in a temperate forest. Sci. Rep-UK. 7, 1267. doi: 10.1038/s41598-017-01260-y 28455492 PMC5430861

[B71] Yang Q. Zhang H. Peng W. Lan Y. Luo S. Shao J. . (2019). Assessing climate impact on forest cover in areas undergoing substantial land cover change using Landsat imagery. Sci. Total Environ. 659, 732–745. doi: 10.1016/j.scitotenv.2018.12.290 31096403

[B72] Zhang J. H. Fu C. B. Yan X. D. Seita E. Hiroshi K. (2002). A global respondence analysis of LAI versus surface air temperature and precipitation variations. Chin. J. Geophys-CH. 45, 662–669. doi: 10.1002/cjg2.28042587375

[B73] Zhang J. Zhao M. Ma R. Feng M. Ding C. Wu L. . (2025). Different impacts of temperature and precipitation on vegetation net primary production of global marsh wetlands. Glob. Planet. Change 252, 104893. doi: 10.1016/j.gloplacha.2025.10489342574925

[B74] Zhao W. Tan W. Li S. (2021). High leaf area index inhibits net primary production in global temperate forest ecosystems. Environ. Sci. pollut. R. 28, 22602–22611. doi: 10.1007/s11356-020-11928-0 33420691

[B75] Zhu Z. Piao S. Myneni R. B. Huang M. Zeng Z. Canadell J. G. . (2016). Greening of the Earth and its drivers. Nat. Clim. Change 6, 791–795. doi: 10.1038/nclimate300437880705

