# Supplementary material for: Divergent impacts of temperature and precipitation changes on leaf area index of deciduous broadleaf and needleleaf forests in the Northern Hemisphere
Source: Front Plant Sci. 2026 Sep 22;17:1919632. doi: 10.3389/fpls.2026.1919632 (PMC13638591; doi:10.3389/fpls.2026.1919632)
Supplement: Supplementary file 1 [file Table1.docx]

Supplementary Material

**Supplementary Table 1.** Results of first-differencing partial correlation analysis between LAI and climatic factors. First-differencing was applied to the 19-year annual time series (2003–2021), yielding 18 year-to-year change observations.

|  | Forest Type | Partial Correlation Coefficient (r) | p-value | Sample Size (n) |
| --- | --- | --- | --- | --- |
| **DF** | LAI vs. P_re_ | 0.049 | 0.856 | 18 |
|  | LAI vs. T_max_ | 0.251 | 0.349 | 18 |
|  | LAI vs. T_min_ | -0.110 | 0.686 | 18 |
| **DBF** | LAI vs. P_re_ | 0.205 | 0.447 | 18 |
|  | LAI vs. T_max_ | 0.674 | 0.004 | 18 |
|  | LAI vs. T_min_ | -0.578 | 0.019 | 18 |
| **DNF** | LAI vs. P_re_ | -0.324 | 0.221 | 18 |
|  | LAI vs. T_max_ | 0.532 | 0.034 | 18 |
|  | LAI vs. T_min_ | -0.337 | 0.201 | 18 |
